# Engineered *Pichia pastoris* for enhanced production of S-adenosylmethionine

**DOI:** 10.1186/2191-0855-3-40

**Published:** 2013-07-27

**Authors:** Venu Kamarthapu, Srinivas Ragampeta, Khareedu Venkateswara Rao, Vudem Dashavantha Reddy

**Affiliations:** 1 Centre for Plant Molecular Biology, Osmania University, Hyderabad 500 007, India; 2 National Centre for Mass Spectroscopy, Indian Institute of Chemical Technology, Hyderabad 500 007, India

**Keywords:** S-adenosylmethionine synthetase, *Pichia pastoris*, Heterologous host, Bioreactor

## Abstract

A genetically engineered strain of *Pichia pastoris* expressing S-adenosylmethionine synthetase gene from *Saccharomyces cerevisiae* under the control of AOX 1 promoter was developed. Induction of recombinant strain with 1% methanol resulted in the expression of SAM2 protein of ~ 42 kDa, whereas control GS115 showed no such band. Further, the recombinant strain showed 17-fold higher enzyme activity over control. Shake flask cultivation of engineered *P. pastoris* in BMGY medium supplemented with 1% L-methionine yielded 28 g/L wet cell weight and 0.6 g/L S-adenosylmethionine, whereas control (transformants with vector alone) with similar wet cell weight under identical conditions accumulated 0.018 g/L. The clone cultured in the bioreactor containing enriched methionine medium showed increased WCW (117 g/L) as compared to shake flask cultures and yielded 2.4 g/L S-adenosylmethionine. In spite of expression of *SAM 2* gene up to 90 h, S-adenosylmethionine accumulation tended to plateau after 72 h, presumably because of the limited ATP available in the cells at stationery phase. The recombinant *P pastoris* seems promising as potential source for industrial production of S-adenosylmethionine.

## Introduction

S-adenosylmethionine (SAM) is a metabolite of the methionine metabolism. In diverse living organisms, it is known to mediate three important metabolic pathways, viz., transmethylation, trans-sulphuration, and aminopropylation (Chiang et al. 1996; Bottiglieri 2002; Fontecave et al. 2004; Roje 2006) SAM is also involved in various other reactions that occur through radical-based catalysis (Grillo and Colombatto 2007). It is known to act as a key physiological compound essential for optimal hepatic function, proper functioning of joints, as well as gastric mucosa protection (Lu 2000; Lu and Mato 2008). As such, it is used extensively in mitigating depression, liver disease and osteoarthritis (Castillo et al. 2005; Papakostas et al. 2003; Mischoulon et al. 2012; Ringdahl and Pandit 2011; Papakostas et al. 2012; Harmand et al. 1987).

However, use of SAM for therapeutic applications is extremely limited owing to its expensive nature. Hence, it is imperative to develop an efficient method for production of SAM. Several attempts have been made to isolate genetic strains accumulating higher concentration of SAM. Yeast strains have been isolated that could accumulate increased concentration of SAM in comparison with other microorganisms (Shiozaki et al. 1984). An efficient positive selection method has been optimized for isolation of SAM accumulating yeast strains with significant increases in SAM content on a dry cell weight basis. Nonetheless, the volumetric yield of SAM is low as most of the strains belonged to *Saccharomyces cerevisiae* and were hardly able to attain high cell density using minimal media (Shobayashi et al. 2006).

The methylotrophic yeast, *Pichia pastoris,* was found to serve as an outstanding host for enhanced production of different proteins (Garg et al. 2012; Lee et al. 2013; Clare et al. 1991; Cregg et al. 1993). *P. pastoris* demonstrated ample potential for high-level expression, efficient secretion and growth rate with very high cell densities. The success of *P. pastoris* system was attributed to its strong, tightly-regulated alcohol oxidase (*AOX1*) promoter (Duff and Murray 1988; Cregg et al. 1989). This promoter can be strongly induced by methanol which also can serves as the main carbon source (Ellis et al. 1985), and repressed by most other carbon sources (Tschopp et al. 1987). Accumulation of methanol leads to cytotoxic effects (Guarna et al. 1997) and induction of the promoter is optimal when the methanol level is kept within growth limiting rates. Another unique feature of this system is that a high cell density can be achieved using the cost-effective, minimal salt medium. Thus far, more than 500 genes of viruses, prokaryotic and eukaryotic microorganisms, plants and humans have been expressed in the *P. pastoris* system (Sreekrishna et al. 1988; Scorer et al. 1994; Cereghino and Cregg 2000; Macauley-Patrick et al. 2005).

Among different SAM synthetase isozymes identified in various microorganisms and animal tissues, SAM synthetase of *S. cerevisiae* exhibited some rare advantages. Expression of *S. cerevisiae SAM2* gene was induced by the presence of excess methionine in the growth medium, while expression of *SAM1* gene was repressed, thereby obviating the problem of product inhibition observed with other SAM synthetases (Chiang and Cantoni 1977; Cherest and Surdin-Kerjan 1981; Thomas et al. 1988).

Attempts have been made to improve the production of SAM in *P. pastoris* using various methods, viz., transformation with *SAM2* gene of *S. cerevisia* (Li et al. 2002); expression of *SAM2* under the control of a constitutive promoter (Yu et al. 2003); co-production of SAM with glutathione by fed-batch fermentation (Lin et al. 2004); and employment of altered feeding strategy in a bioreactor (Hu et al. 2009). *SAM2* gene was expressed and cystathione-β-synthase gene was knocked out from *P. pastoris* for production of SAM (He et al. 2006; Yu and Shen 2012). Enhanced accumulation of SAM was achieved by manipulating the culture conditions like increased oxygen levels and nitrogen source (Chen et al. 2007; Zhang et al. 2008a; Zhang et al. 2008b; Chu et al. 2013). Earlier studies reported elevated intracellular production of SAM using recombinant *P. pastoris;* however, these reports did not confirm the expression of the enzyme by SDS-PAGE analyses, and molecular characterization of the accumulated SAM using MS/MS method.

In the present study, we have cloned *SAM2* gene from *S. cerevisiae,* introduced into the genome of *P. pastoris,* and optimized the conditions for cultivation of the engineered *P. pastoris* in the methionine-enriched medium for enhanced accumulation of SAM in shake flask. Furthermore, the recombinant *P. pastoris* has been cultivated in a 14 L bioreactor to increase the wet cell weight and SAM accumulation. The accumulated SAM has been identified, quantified and characterized by HPLC and LC-MS/MS analyses.

## Materials and methods

### Amplification and cloning of *S. cerevisiae* SAM synthetase gene

*S. cerevisiae* strain INVSc1 (invitrogen) was grown in the YPD medium (1% Peptone, 2% Yeast extract, 2% dextrose) overnight at 30°C at 220 rpm. Gnomic DNA was isolated as per the protocol of Sambrook and Russel (2001). Amplification of SAM synthetase coding sequence was carried out employing the primers, 5’GCGCGGATCCACCATGGCCAAGAGCAAAACTTTC 3’ and 5’GCGCGAATTCTTAAAATTCCAATTTCTTTGG 3’. Amplified *SAM* sequence was digested with *Bam*H I and *Eco*R I and cloned in to pPIC3.5 K vector. Recombinants were identified by restriction analysis of plasmid DNA with *Bam*H I- *Eco*R I, *Sac* I, *Nco* I and *Nde* I and were confirmed by sequencing of both the strands.

### Transformation of *Pichia pastoris* by electroporation method

A single colony of *P. pastoris* (GS115) was inoculated into 5.0 ml of YPD medium in a 50 ml conical flask and grown at 30°C, 280 rpm for overnight. Next day, 500 ml of YPD medium was inoculated with 0.5 ml of overnight culture and grown at 30°C, 280 rpm until the cell density reached OD_600_ =1.5. The culture was centrifuged at 5000 xg for 5 min at 4°C, and the pellet was re-suspended in 500 ml of ice-cold sterile water. The cells were centrifuged again, and then re-suspended the pellet in 250 ml of ice-cold sterile water. The centrifugation was repeated as above and re-suspended the pellet in 20 ml ice-cold 1.0 M sorbitol. These cells were later centrifuged at 5000 x g for 5 min at 4°C, and the pellet was re-suspended in 1.0 ml of ice-cold 1.0 M sorbitol. From the suspension, 80 μl of cells were mixed with 20 μg of *Sac* I linearized pPIC3.5 K-*SAM2*/20 μg pPIC3.5 K DNA (vector alone) and the same were transferred into ice-cold 4 mm electroporation cuvette, and the cuvettes with cells were incubated on ice for 5 min. The various parameters of the pulse generator (Electro Cell Manipulator ECM 600 BTX Electronic Genetics, San Diego, CA) were setup as follows: high-voltage mode, resistance 129 Ohms, charging voltage of 1.5 kV, estimated field strength of 7.5 kV/cm and pulse length of 5 msec. Cuvette was placed in the electroporation chamber, connected to the pulse generator and subjected to two pulses; followed by immediate addition of 1.0 ml of ice-cold 1.0 M sorbitol to the cuvette. The cuvette contents were transferred to the sterile microcentrifuge tube; from this an aliquot of 500 μl was plated on RDB medium (1.0 M sorbitol, 2% dextrose, 1.34% of yeast nitrogen base with ammonium sulphate, 0.4 mg/L biotin, 2% bactoagar and 0.005% of each L-glutamic acid, L-methioninie, L-lysine, L-leucine, and L-isoleucine) lacking Histidine. The plates were incubated for 3 to 5 days at 29°C until His^+^ recombinant colonies were developed.

### Screening of recombinant (His^+^) colonies with multiple inserts using antibiotic G418

Two hundred microlitres of YPD medium was placed in each well of the microtiter plate (96 wells) under sterile conditions. Each well was inoculated with a single His^+^ transformant using a sterile toothpick and stirred to resuspend the cells. The microtiter plates were covered with sterile aluminum foil and incubated at 30°C for 2 days. Later, sterile microtiter plates were used and added with 190 μl of YPD medium in each well, and inoculated with 10 μl of the culture from the first set of microtiter plates. Second set of plates were marked and oriented to keep track of the wells. The plates were covered and incubated at 30°C for overnight. Next day, a third set of microtiter plates were sub-cultured as described above. Again the plates were incubated at 30°C for overnight. From the third set of plates, 10 μl of culture from each well was spotted on YPD plates containing 0, 0.25, 0.5, 1.0 and 2.0 mg/ml of G418. Plates were marked properly to track all the clones at different concentrations of G418. Plates were incubated at 29°C, and checked after 2^nd^, 3^rd^, 4^th^ and 5^th^ day for G418 resistant clones.

### Colony PCR screening for presence of *SAM2* gene in recombinant clones

From 2 mg/ml G418 plate, a single colony was picked up and re-suspended in the 10 μl of sterile water in the micro centrifuge tube. To this, 5.0 μl of 10 U/μl solution of Zymolase was added and incubated at 30°C for 15 min. The tubes were then immersed in the liquid nitrogen for 2 min. From the resulting lysate, 5.0 μl was taken and the polymerase chain reaction was set up by adding 50 picomoles each of 5’ AOX and 3’ AOX primers. Introduced *SAM2* gene was confirmed by the PCR employing gene specific primers. The clone has been designated as GS115-*SAM2.* After confirming clones, glycerol stocks were prepared and stored at –70°C.

### Culturing of recombinant *P pastoris* (GS115-*SAM2*) in shake flask

A single colony of GS115-*SAM2*/control GS115 transformed with vector alone, grown on YPD agar plate, was inoculated in 5.0 ml of YPD medium and grown at 30°C, 280 rpm for overnight, and was used as inoculum. All the shake flask experiments were carried out in 250 ml conical flasks (25 ml working volume). One percent of inoculum (v/v) was transferred into BMGY medium consisting of 1% yeast extract, 2% peptone, 1.34% of Yeast nitrogen base with ammonium sulphate and without amino acids, 4% of glycerol, 100 mM potassium phosphate buffer (pH 6.0) and 0.4 mg/L biotin with 1% L-methionine. Culture was grown at 30°C, 280 rpm until culture reached OD_600_ = 3.0. Methanol (1% v/v) was added after every 24 h to the culture and induction was continued for 4 days. Two milliliters of culture was withdrawn from each flask per every 24 h and centrifuged at 5000 x g for 3 min. The pellet was stored at –70°C until processing. These samples were used to analyze expression of heterologous SAM synthetase and accumulation of SAM to determine the optimal harvest time and selection of potential recombinant *P. pastoris* with maximum SAM accumulation. The cultures were centrifuged at 5000 x g for 5 min, and the pellet was stored at –70°C.

### Fermentation of recombinant *P. pastoris* in bioreactor

Fermentation was carried out in a 14 L bench top fermentor Bioflo 2000 (New Brunswick Scientific, Edison, NJ, USA). Seed culture of recombinant *P. pastoris* for fermentation was initiated from the fresh glycerol stock and inoculated into 250 ml conical flask containing YPD medium (50 ml working volume) and grown for 24 h. Four percent inoculum was used for inoculation of a 14 L fermentor containing 6.0 L of BMGY medium containing 1% L-methionine. The aeration rate was initially maintained at 0.5 min^-1^ VVM and then increased to 1.0 min^-1^ VVM when required. Dissolved oxygen was kept above 20% by controlling the agitation between 400 and 600 rpm. The pH of the medium was controlled at 5.8 by adding ammonia solution. Temperature was maintained at 29°C. Foaming was controlled by addition of 1.0 ml of antifoam agent from Sigma Aldrich. Cells were grown until glycerol was completely depleted (28 h). Then, methanol feed was initiated and maintained at 1% throughout the fermentation. Samples were collected after every 12 h for SAM synthetase expression, SAM accumulation and biomass assay. The samples were centrifuged at 5000 x g for 5 min and the pellet was stored at –70°C for SDS-PAGE analysis. At the end of the fermentation, culture was centrifuged at 5000 x g for 10 min and the pellet was stored at –70°C.

### HPLC analysis of SAM

Cells were collected by centrifugation and washed with water. SAM was extracted from the cells with 1.5 M perchloric acid for 1 h. The supernatant was collected after centrifugation at 8000 xg for 5 min. A 20 μl of the extracted sample was analyzed by HPLC (Shimadzu, Japan) using a Hypersail SCX column (4.6 mm × 250 mm, 5 μm). The mobile phase consists of 100 mM ammonium formate (pH adjusted to 4.0 with formic acid). SAM accumulation was monitored at λ = 254 nm, and was identified and quantified using the standard SAM (Sigma, USA).

### SDS-PAGE analysis and determination of enzyme activity

The cell pellets from 1 ml culture were resuspended in 500 μl of ice-cold breaking buffer (50 mM sodium phosphate, 1.0 mM PMSF, 1.0 mM EDTA, 5% glycerol, pH 7.4). An equal volume of acid-washed glass beads (size, 0.5 mm), vortexed for 30 seconds, then incubated on ice for 30 seconds, repeated this step for a total of 8 cycles. After centrifugation at 10000 x g for 5 min at 4°C, the supernatant was transferred to a microcentrifuge tube and used for the analysis. From the above supernatant 50 μl was mixed with 50 μl 2X gel loading dye. From this 10 μl was loaded onto 12% SDS-PAGE and subjected to electrophoresis. The gel was stained with Coomassie blueR-250 and destained using solution (30:10:60 of methanol, acetic acid and water) till the protein bands were apparent on a clear background. Enzyme activity was determined as per the protocol of Kamarthapu et al. (2008).

### LC-MS/MS analysis

A high performance liquid chromatography (HPLC) system Agilent 1200 series (Agilent Technologies, USA) was interfaced to a Q-TOF mass spectrometer (Agilent Technologies, USA). Chromatographic separation was achieved using an Eclipse XDB-C18, 5.0 μm particle size analytical column (4.6 × 150 mm) from Agilent, at a flow rate of 1.0 ml/min with isocratic mobile phase (100 mM ammonium formate, pH was adjusted to 4.0 with formic acid). The elution profile was monitored by absorbance at 254 nm using G1315D multiple wavelength UV/VIS detector. After LC, sample enters in to the Mass spectrometer (Agilent 6510 series Classic G6510A Q-TOF LC/MS, Agilent Technologies, USA). Electrospray ionization (ESI) mass spectra were obtained by setting electrospray interface in positive-ionization mode with the skimmer potential 65 V, capillary exit 80 V, and a source of temperature at 300°C, to obtain maximum abundance of the standard sample in full scan spectra (100-1000 Da, 10 full scan/s). Nitrogen was used as a drying (6.0 L/min) and nebulizing gas (30 psi). Total ion chromatogram and mass spectra were processed using Agilent Mass Hunter workstation Software.

## Results

Nucleotide sequence analysis of recombinant plasmid confirmed the presence of SAM2 (GenBank accession number KF142161). The recombinant plasmid pPIC3.5 K-*SAM2* linearized with *Sac* I was introduced into *P. pastoris* strain GS115 by electroporation method. Following electroporation cells were plated onto selection medium-lacking Histidine. Two hundred and fifty colonies were observed after 5 days of plating. Further, these clones were subjected to antibiotic selection on YPD medium containing varied concentrations (0.25-2.0 mg/ml) of G418. Out of 250 clones, 208 could exhibit growth on 0.25 mg/ml G418 selection medium; of these 208 clones, 127 of them showed growth on 0.5 mg/ml G418. From these 127 clones, 56 clones exhibited growth on 1.0 mg/ml G418 containing medium, whereas only 15 clones were able to grow on 2.0 mg/ml G418 containing medium.

**Figure 1:**
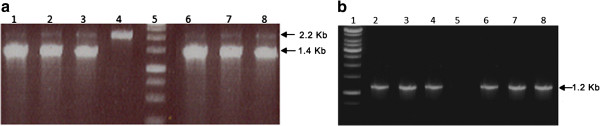
**Confirmation of transformed clones of *P. pastoris *by colony PCR with AOX and gene specific primers. a**: Amplification with AOX primers. Lanes 1, 2, 3, 6, 7 and 8: Clones exhibiting an amplified thick band of ~ 1.4 Kb *SAM2* gene and a faint band of 2.2 Kb *AOX* gene. Lane 4: Untransformed colony exhibiting amplified band of 2.2 Kb *AOX* gene. Lane 5: DNA Marker (1.0 Kb ladder) showing bands of 250 bp, 500 bp, 750 bp, 1.0 Kb, 1.5 Kb, 2.0 Kb &2.5 Kb. **b**: Amplification with gene specific primers of *SAM2* gene. Lane 1: DNA Marker (1.0 Kb ladder) showing bands of 750 bp, 1 Kb, 1.5 Kb, 2.0 Kb, 2,5 Kb, 3.0 Kb, 3.5 Kb, 4.0 Kb, 5.0 Kb and 6.0 Kb. Lanes 2, 3, 4, 6, 7 and 8: Clones exhibiting amplified band of ~ 1.2 Kb corresponds to *SAM2* gene. Lane 5: Untransformed GS115 strain showing no amplification.

His^+^ transformants, selected on YPD plates containing 2.0 mg/ml G418, were analyzed for the presence of *SAM2* gene using colony PCR. PCR analysis revealed the presence of a faint band of ~ 2.2 Kb corresponding to the *AOX1* gene, and a 1.4 Kb amplification product composed of *SAM2* gene and 220 bp sequence from the pPIC3.5 K vector (Figure 1a). The untransformed *P. pastoris* GS115 resulted in a bright band of ~ 2.2 Kb corresponding to the *AOX1* gene (Figure 1a). PCR analysis using the *SAM2*gene specific primers resulted in ~1.2 Kb amplification product corresponding to *SAM2* gene, while untransformed GS115 failed to show the band (Figure 1b).

### Expression of recombinant SAM synthetase and production of SAM in the shake flask cultures

**Figure 2:**
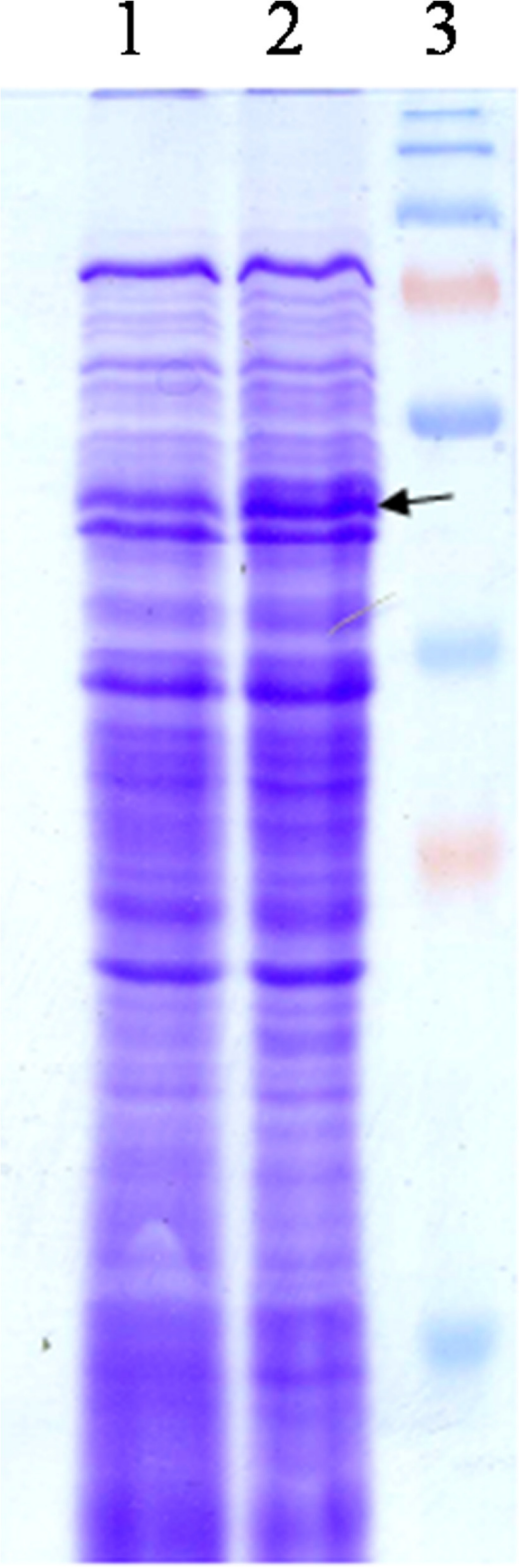
**SDS-PAGE analysis showing expression of *Saccharomyces *SAM synthetase in *P. pastoris*.** Lane 1: Control, GS115 transformed with vector alone showing no expression of SAM synthetase. Lane 2: Recombinant *P. pastoris* showing induced band of SAM synthetase (~ 42 kDa). Lane 3: Pre-stained marker.

**Figure 3:**
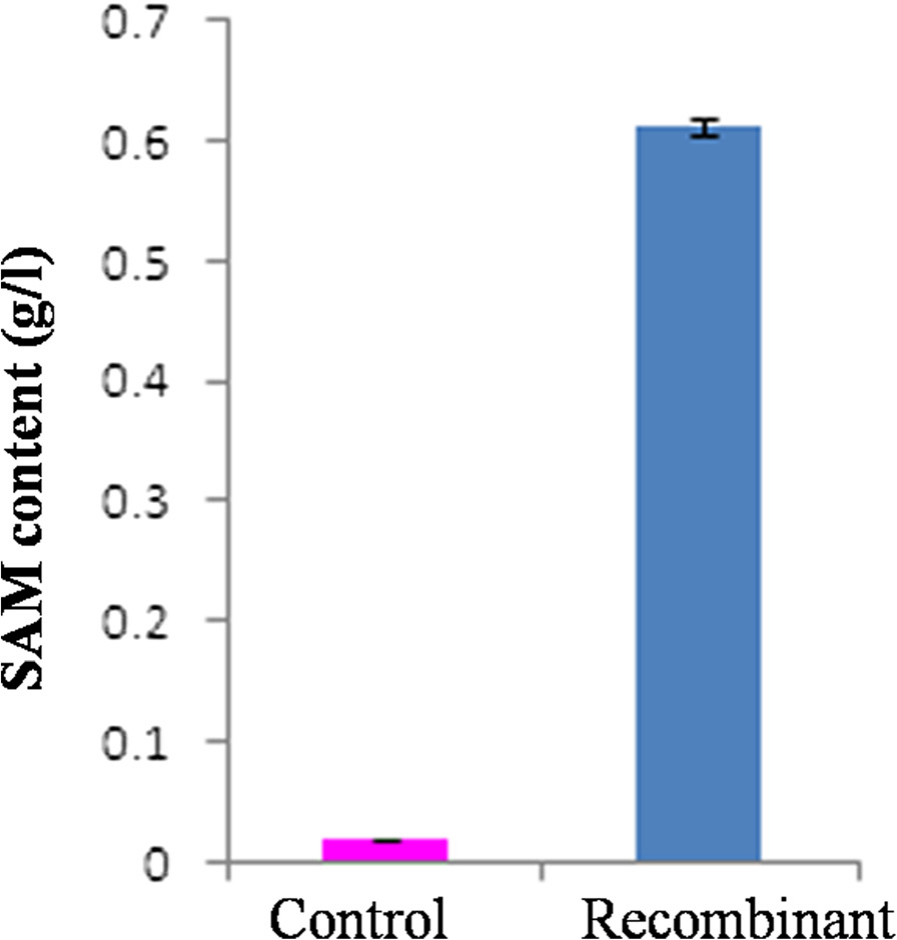
**Recombinant *P. pastoris *GS115 expressing *SAM2 *gene when cultured in shake flask accumulated 33-fold higher SAM content as compared to Vector alone transformed strain of GS115.**

Induction of recombinant *P. pastoris* with 1% methanol at log phase of OD_600_ = 3.0 and pH of 6.0, resulted in the expression of *SAM2* protein of ~ 42 kDa, whereas control GS115 showed no such band (Figure 2). All the 15 clones, expressing *SAM2* gene, exhibited enhanced accumulation of the SAM, when compared to the control GS115 transformed with the vector alone. One of the high expressing transformant was selected for further studies, which yielded wet cell weight (28 g/L) and SAM (0.6 g/L or 0.195 g/g DCW). Whereas, the control (transformant with vector alone), under the identical culture conditions with similar wet cell mass, produced 0.018 g/L SAM (Figure 3) or 0.0054 g SAM/g DCW. Further, the clone expressing *SAM*2 gene showed more than 17 fold higher enzyme activity as compared to control.

### Production of SAM in the bioreactor

**Figure 4:**
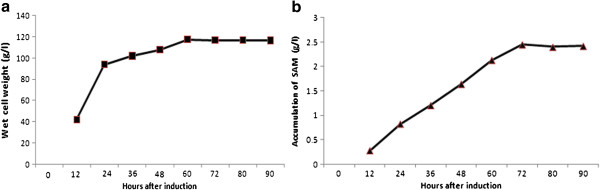
**Gain in wet cell weight and accumulation of SAM in the recombinant *P. pastoris *GS115 cultured in bioreactor at different time points after induction with methanol. a** Wet cell weight **b** SAM content.

The clone GS115-*SAM2* was cultivated in the bioreactor for production of SAM. The clone was initially grown on glycerol as the sole carbon source for ~28 h. Then feeding with methanol was continued for a further period of 90 h. SDS-PAGE analysis of the recombinant *P. pastoris* revealed ~ 42 kDa SAM synthetase protein, from 12-90 h induction, with maximum expression of recombinant SAM synthetase from 48-60 h. Accumulation of SAM in the bioreactor also increased gradually from 12 to 72 h and there was no increase in the accumulation after 72 h (Figure 4b). At the end of cultivation of the recombinant *P. pastoris*, wet cell weight of 117 g/L was achieved (Figure 4a). The recombinant *P. pastoris* cultured in the bioreactor, enriched with methionine could accumulate 2.4 g/l SAM (Figure 4a) or 0.021 g/g WCW or 0.186 g/g DCW.

### LC-MS/MS analysis of the extract of recombinant *P. pastoris*

**Figure 5:**
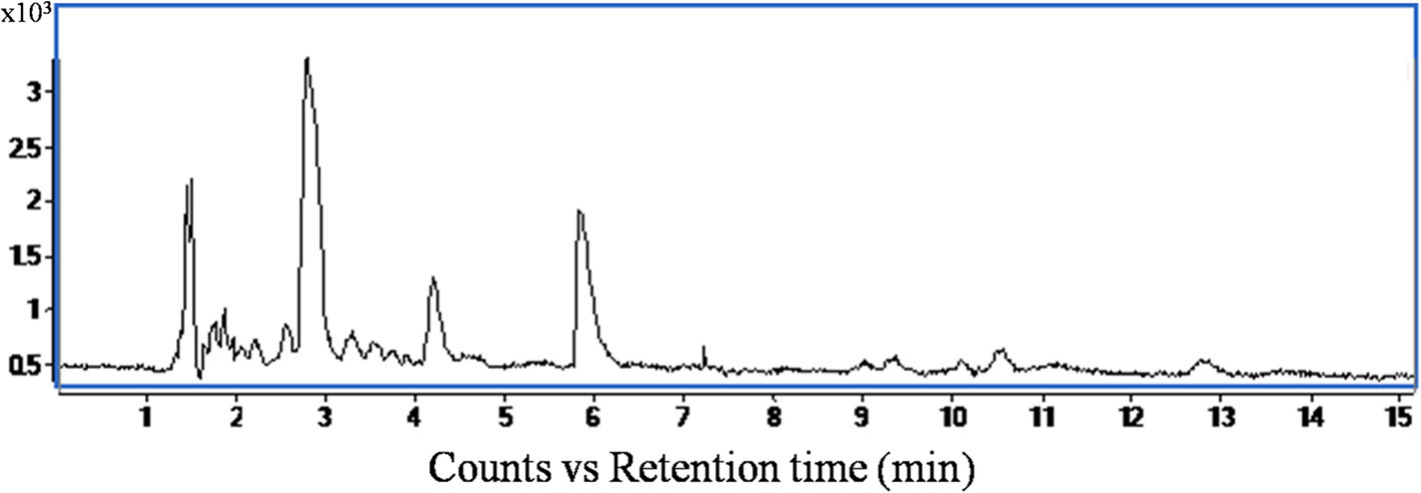
Total ion chromatogram using positive ESI-MS detection.

**Figure 6:**
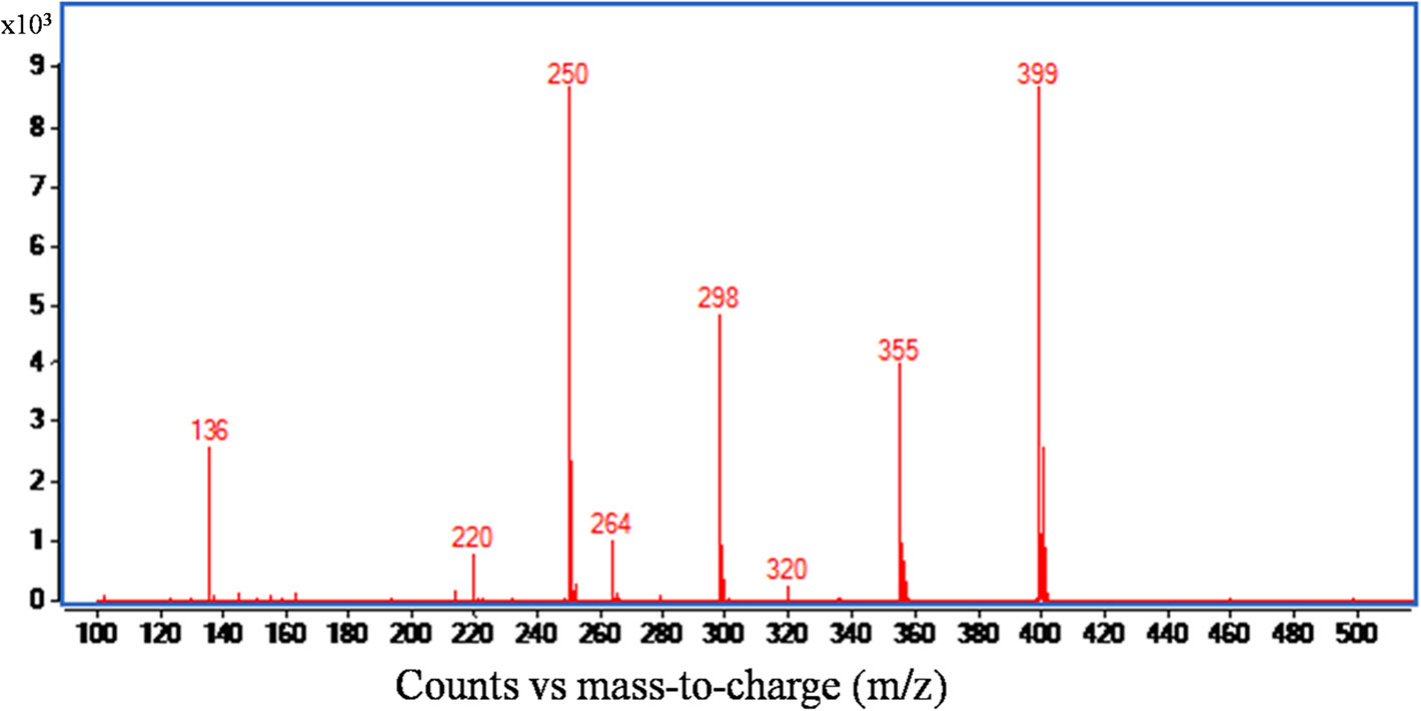
**ESI mass spectrum corresponds to t**_**R **_**at 2.7 min.**

**Figure 7:**
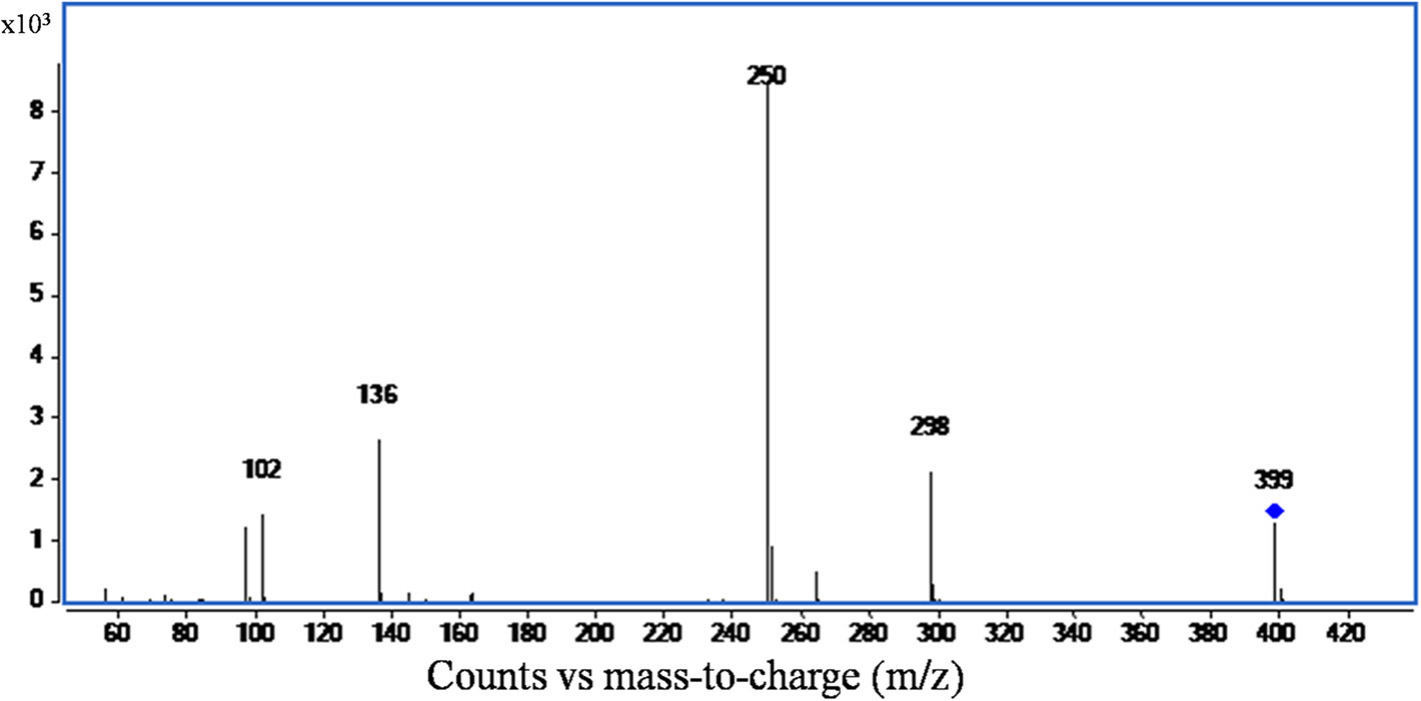
**CID MS/MS spectrum of *m/z *399 at 12 eV.**

LC/MS analysis of acid-extracted fermented cells exhibited distinct chromatographic peaks at retention times (t_R_) of 1.4, 1.5, 2.7, 4.2 and 5.8 minutes (Figure 5). The ESI mass spectrum of the chromatographic peak eluted at a t_R_ of 2.7 minutes (Figure 6), and the spectrum showed [M]^+^ion at *m/z* 399 as the base peak. The spectrum also showed moderately abundant fragment ion peaks at *m/z* 355, 298, 136 and highly abundant ion peak at *m/z* 250. The collision induced dissociation (CID) tandem mass spectrometry (MS/MS) of m*/z* 399 at 12 eV disclosed characteristic signals at *m/z* 298, *m/z* 250, *m/z* 136, and *m/z* 102 (Figure 7).

## Discussion

Biosynthesis of SAM is catalyzed in cytosol of cell by SAM synthetase using ATP and L-methionine as the substrates. SAM presents a unique feature in which all its constituents participate in diverse biochemical reactions (Fontecave et al. 2004). SAM is being used as a drug to treat liver diseases, depression, osteoarthritis, fibromyalgia, and Alzheimer’s disease (Lu 2000). In view of therapeutic importance of SAM, cost effective production of biologically active (*S, S*) form in large quantities is the need of the hour. as developed by the overexpression of *S. cerevisiae* SAM synthetase. The recombinant *P.* Various strains belonged to *S. cerevisiae* (Shiomi et al. 1990), *Kluyveromyces lactis* (Mincheva et al. 2002) and *S. sake* (Shiozaki et al. 1984) with high productivity of SAM have been isolated. However, low culture densities achieved in the minimal medium was not suitable for an industrial production of SAM. In the present investigation, an engineered *P. pastoris* was cultivated in shake flask and bioreactor. As compared to control the engineered strain showed enhanced accumulation of SAM. Further, the accumulated SAM was characterized at molecular level by LC-MS/MS analysis.

Recombinant plasmid pPIC3.5-*SAM2* linearized with *Sac* I enzyme and electroporated into *P. pastoris* GS115, showed growth on histidine deficient medium, thus suggesting the transformed nature of colonies. Most of the His^+^ clones (~ 83%) exhibited growth on medium containing antibiotic G418, indicating the stability of the transformants. All the His^+^ clones resistant to G418, when subjected to PCR analysis using *AOX1* primers, gave a faint amplification product (~ 2.2 Kb) corresponding to the native *AOX1* gene and a bright amplification product (~1.4 Kb) comprising of *SAM2* gene, implying stable integration of recombinant plasmid, containing *SAM2,* gene by homologous recombination at *AOX1* in the genome of *P. pastoris.* All these clones exhibited Mut^+^ phenotype. In an earlier study, it was found that amplification of both bands corresponding to the insert and *AOX1* gene was used as indication of Mut^+^ phenotype, while Mut^s^ was distinguished by the lack of amplification pertaining to native *AOX1* gene (Linder et al. 1996). Transformed clones exhibited varied levels of resistance to antibiotic G418. The variation observed for resistance to antibiotic is attributable to the variable copy number of Kanamycin resistance gene present in different transformants. Of the 250 His^+^ clones only 15 of them exhibited growth on 2 mg/ml G418 containing medium, suggesting that these transformants might have acquired 4-7 copies of kanamycin resistance gene. Earlier studies, demonstrated a strong correlation between the integrated copies of the Kanamycin resistant gene into the *P. pastoris* genome and the degree of resistance to antibiotic G418. Further, it was established that *P. pastoris* transformants exhibiting tolerance to 0.25, 0.5, 1.0 and 2.0 mg/ml G418, showed the integration of 1 to 2, 3 to 4 and 4 to 7 copies, respectively, of kanamycin resistance gene (Scorer et al. 1994; He et al. 2006). Further, the level of resistance to antibiotic (2 mg/ml) revealed by different transformants amply testify the integration of 4-7 copies of *SAM2* gene as these two genes are linked in the recombinant plasmid employed for genetic transformation.

In the shake flask experiment, SDS-PAGE analysis of engineered *P. pastoris* induced with methanol exhibited ~ 42 kDa protein band, while no such band was observed in the *P. pastoris* transformed with vector alone, confirming the expression of *SAM2* gene in the heterologous host. As compared to control, engineered strain showed 17 fold higher enzyme activities than control strain, confirming the expression of *SAM2* gene. Recombinant clone, cultured in L-methionine enriched medium, when subjected to methanol induction, accumulated 33-fold more SAM (0.6 g/l) than the *P. pastoris* transformed with vector alone*,* in shake flasks culture, indicate the functional nature of heterologus SAM synthetase. The enhanced accumulation of SAM in the recombinant *P. pastoris* is attributable to the enriched substrate methionine and high-level expression of heterologus SAM synthetase insensitive to product inhibition, contributing to the increased SAM synthesis. In an earlier study, culturing of 9 different species of *Saccharomyces* in medium supplemented with L-methionine showed improved intracellular accumulation of SAM in these species. *S. sake* K-6 exhibited maximum accumulation of 12.6 μmol SAM (Shiozaki et al. 1989). Enrichment of culture medium with Precursor L-methionine increased the metabolic flux of SAM in various microorganisms (Shiozaki et al. 1984).

The engineered *P. pastoris* when cultivated in the bioreactor, promoted the harvest of ~ 4.5-fold increased WCW (117 g/l) in comparison with the shake flask cultures (28 g/l). The significant increase observed in the production of WCW is ascribable to the optimum dissolved oxygen, nutrient balance and pH (5.8) maintained throughout the fermentation. Compared to shake flask cultures, cultivation of *P. pastoris,* expressing *SAM2* gene, in the bioreactor produced 4-fold increases in SAM (2.4 g/l). Enhanced accumulation of SAM by the recombinant *P. pastoris,* when cultured on L-methionine enriched medium, in the bioreactor is mainly attributable to the increased WCW of *P. pastoris*. Accumulation of SAM in the bioreactor increased gradually from 12 to 72 h after methanol induction. SDS-PAGE analysis revealed that the expression of SAM2 was maximum after 48 to 60 h of induction with methanol. Inspite of the expression of *SAM2* gene up to 90 h, SAM accumulation tended to plateau after 72 h, presumably because of the limited ATP available in the cells at stationary phase.

The acid extraction of fermented cells was analyzed and SAM was quantified by the HPLC. Molecular characterization of the SAM was carried out by MS/MS analysis and the ESI mass spectrum of the chromatographic peak, eluted at a t_R_ 2.7 minutes, disclosed [M]^+^ion at *m/z* 399 as the base peak corresponding to the SAM. As SAM is positively charged because of the tertiary sulphur atom, hence, no [M + H]^+^ ion was observed. The collision induced dissociation, tandem mass spectrometry of *m/z* 399 showed signals at *m/z* 298 and at *m/z* 250 indicating the loss of 2-amino 3-butenoic acid and methionine, respectively. The signals at *m/z* 136 and at *m/z* 102 represent protonated adenine and 2-amino 3-butenoic acid, respectively. These results confirm that the *m/z* 399 peak eluted at t_R_ 2.7 minutes corresponds to SAM, and is in conformity with the established mass spectroscopic profiles of SAM (Struys et al. 2000).

In conclusion, *S. cerevisiae SAM2* gene was successfully introduced in to the genome of *P. pastoris* under the control of *AOX1* promote*r.* In shake flask cultivation, the engineered *P. pastoris* could produce a 33-fold increase in SAM accumulation than the control *P. pastoris* transformed with the vector alone. In bioreactor, the engineered *P. pastoris* was grown to high cell densities and produced a four-fold increase in SAM accumulation than cultivation in the shake flask. The overall results amply demonstrate the overexpression of *SAM2* gene in heterologus host *P. pastoris* lead to enhanced production of SAM when cultured in methionine enriched medium. The recombinant *P. pastoris* seems promising as potential source for industrial production of SAM.

## Competing interests

The authors declare that they have no competing interests.
